# Frequency-specific neural abnormalities in congenital prosopagnosia revealed by magnetoencephalography during face perception

**DOI:** 10.1038/s41598-025-16958-7

**Published:** 2025-08-28

**Authors:** Yutaka Kato, Yuichi Takei, Masakazu Sunaga, Mika Konishi, Masaru Mimura, Seiichiro Jinde

**Affiliations:** 1 Tsutsuji Mental Hospital, Kokuwabara 1505, Tatebayashi, Gunma Prefecture 374-0037 Japan; 2 Department of Psychiatry and Neuroscience, Gunma University Graduate School of Medicine, Maebashi, Gunma Prefecture 371-8511 Japan; 3 Department of Neuropsychiatry, Keio University School of Medicine, Tokyo, 160-8582 Japan; 4 Center for Preventive Medicine, Keio University, Tokyo, 106-0041 Japan

**Keywords:** Congenital prosopagnosia, Magnetoencephalography, Face perception, Neural oscillations, Sensor-level analysis, Visual processing (6words/6), Diagnostic markers, Neurophysiology, Disease model, Cognitive control, Inhibition-excitation balance

## Abstract

Congenital prosopagnosia (CP) is characterized by lifelong impairment in face recognition despite intact basic visual processing. While previous studies have demonstrated preserved “core” face processing with disrupted information propagation to “extended” regions, the temporal dynamics of these deficits remain unclear. Here, we used magnetoencephalography (MEG) during a seeing-as-face task to investigate frequency-specific neural mechanisms in three individuals with CP compared to seventeen healthy controls. By presenting identical visual stimuli perceived either as abstract patterns (Non-Face condition) or schematic faces (Face condition), we isolated face-specific cognitive processes while controlling for low-level visual processing. CP showed preserved early face detection (M120) but exhibited enhanced alpha-band (8–13 Hz) activity in right anterior temporal sensors in the 300–450 ms time window (p = 0.046, cluster-corrected) during face processing – the sole finding surviving rigorous statistical correction. This abnormality was specific to Face conditions and suggests disrupted information integration between core and extended face processing networks. The temporal dissociation between preserved early detection and impaired later processing supports conceptualizing CP as a disconnection syndrome. Our preliminary findings suggest potential frequency-specific neural patterns of face processing deficits in CP, providing neural signatures that may warrant investigation in larger studies.

## Introduction

Congenital prosopagnosia (CP) is characterized by lifelong impairment in face recognition despite intact basic visual processing and cognitive functions^1^. Unlike acquired prosopagnosia, which results from brain damage to the inferior occipitotemporal cortex^2,3^, CP occurs without obvious brain lesions^4,5^. Individuals with CP exhibit normal sensory vision, visual knowledge, cognitive, and intellectual functions, but selective deficits in distinguishing human faces, often developing compensatory strategies using non-facial features such as hairstyle, voice, or gait^6,7^.

Face processing involves a complex network comprising “core” and “extended” regions with bidirectional connections^8–10^. The core system includes the fusiform face area (FFA)^11–13^, occipital face area (OFA)^14,15^, and posterior superior temporal sulcus (pSTS) in the ventral occipitotemporal cortices. Initially, face detection occurs in the OFA, extracting face-arranged shapes from visual input^16–18^. Subsequently, holistic/configural processing integrates these features into a single global representation^19,20^ at 170 ms in the FFA^21,22^ by configuring all parts together rather than independently^23,24^. Single-unit recordings in macaques have shown that initial activation of face-selective neurons in ventral occipitotemporal cortices and pSTS is followed by the subsequent firing representing finer information such as a facial expression or individual identity^25,26^. This delayed face-selective processing occurs in the anterior temporal lobe, associated with face identity processing in macaques^27^ and humans^28^. Face-selective patches in ventral anterior temporal lobes (vATL) are interconnected with the “core” network as part of the “extended” system, playing a crucial role in facial identification^29,30^.

Neuroimaging studies have revealed that CP shows largely preserved function in core processing regions. fMRI studies have indicated that FFA, OFA, and pSTS function is intact in CP, suggesting disrupted information propagation beyond the “core” regions^31^. Connectivity research has supported the disconnection hypothesis of CP by demonstrating decreased white matter integrity in pathways linking ventral occipitotemporal and anterior temporal cortices^32–34^. However, neurophysiological studies using EEG and MEG have demonstrated heterogeneous impairments in face recognition, memory, and learning^35,36^, even in face-selective responses (N170/M170) evoked around 170 ms after stimulus onset^37,38^. This suggests partial compensation through enhanced feature processing in the left hemisphere^39^. Due to this heterogeneous presentation^40^, the neural basis of CP remains unclear, though we hypothesize that face identification impairments originate from vATL dysfunction.

To address these issues, we investigated frequency-specific responses during face processing using MEG, which provides excellent temporal and spatial resolution. We employed the"seeing-as-face"(SAF) task^18^, where identical visual stimuli are perceived either as abstract patterns or faces across two blocks. While we initially hypothesized early-stage face detection deficits in CP, our findings revealed preserved early detection (M120) with abnormalities in later face identification processes, consistent with established understanding that CP primarily affects face recognition rather than face detection. This approach allows extraction of face-selective components while controlling for low-level visual properties, enabling identification of aberrant sensor-level abnormalities in CP.

## Results

**Fig. 1:**
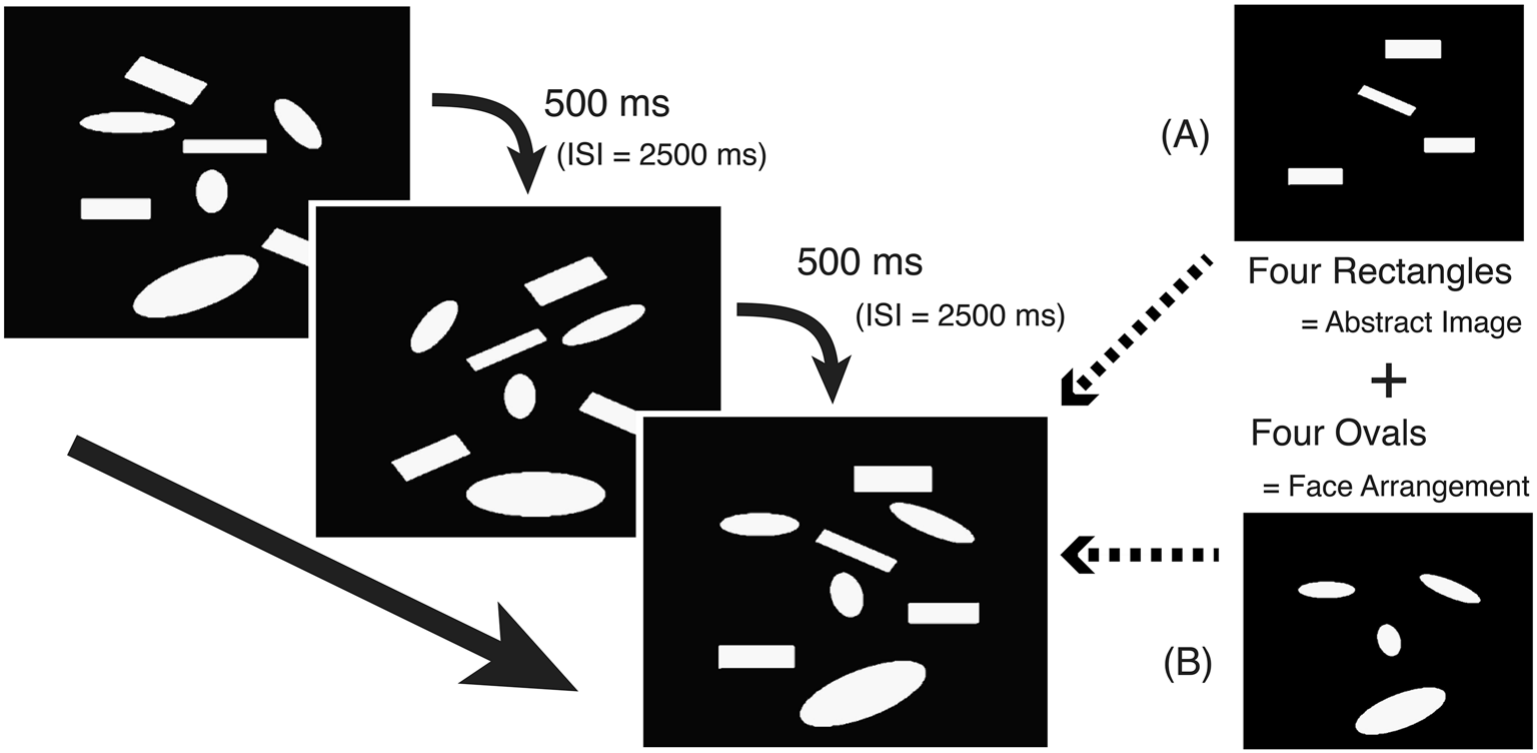
Examples of the presented visual images. The images consisted of (**A**) four rectangles and (**B**) four ovals. The rectangles formed an abstract image, while the four ovals formed as a face. The subjects were instructed to determine whether the target image (first as Non-Face condition gazing rectangles of abstract image; second as Face condition gazing ovals forming face) were the same as those in the previously presented image in terms of its size and arrangement. When the subjects paid attention to the rectangles, only an abstract image was perceived. Next, after being instructed that the four ovals formed a face by ignoring rectangles, the subjects gradually recognized the arrangement of the four ovals as a face. The two blocks were administered successively using the same figure sets in the same sequence, only subjective perception differing.

Using MEG during the seeing-as-face task^18^ (Fig. 1), we compared neural responses between three individuals with congenital prosopagnosia (CP) and seventeen healthy controls (HCs). By presenting identical visual stimuli across Face and Non-Face conditions, we examined potential frequency-specific differences in CP during face processing. The results revealed preserved early face detection (M120) components in both groups. After applying appropriate statistical corrections including cluster-based permutation testing for multiple comparisons, we observed a single statistically significant difference only in alpha-band (8–13 Hz) activity in the right anterior temporal channel-cluster in the 300–450 ms time window during Face conditions (p = 0.046, permutation corrected). All other frequency bands and channel locations showed no significant between-group differences after correction for multiple comparisons. Given the extremely limited sample size (n = 3 CP participants), these findings should be interpreted as preliminary observations requiring replication in larger cohorts.

### Grand average waveforms and frequency properties: fig. 2

**Fig. 2:**
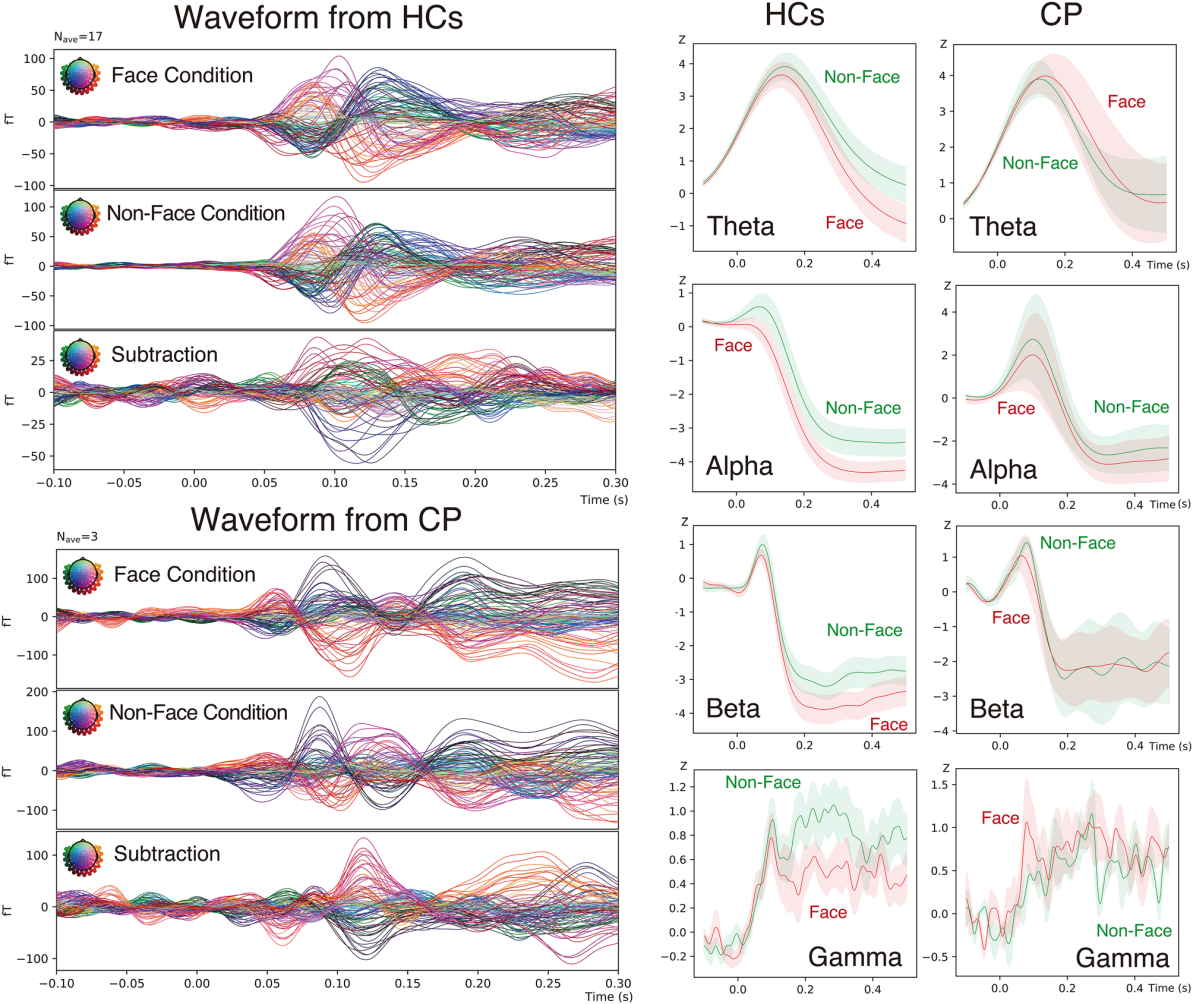
Grand average waveform from HCs and CP. The left panels show the grand average waveforms from seventeen healthy control (HCs: Upper) and three individuals with CP (CP: Lower). The responses measured under Face condition, Non-Face condition, and their Subtraction (Face minus Non-Face) are presented from top to bottom. The X-axis shows the latency from −100 ms to 300 ms. The Y-axis shows the amplitudes described as fT (Face and Non-Face: ± 100 fT; Subtraction: ± 50 fT for HCs, and ± 100 fT for CP) with their colors according to the channel location. The face and non-face conditions elicited several components, which were diminished by subtraction to enhance the component at the latency of 120 ms (M120), which was comparable between HCs and CP. In both groups, M120 components originated from bilateral temporal scalp locations according to their line colors. The right panels show the response power across conditions for HCs (left) and CP participants (right) within theta (4-7 Hz), alpha (8-13 Hz), beta (14-39 Hz), and gamma (40-98 Hz) frequency ranges. Green lines represent the Non-Face condition and red lines represent the Face condition, with thin lines indicating ± 1 standard error of the mean (SEM). In HCs, Face condition responses generally showed attenuation compared to Non-Face responses after approximately 100 ms latency across all frequency ranges. In contrast, CP participants exhibited nearly identical responses between conditions.

Grand average waveform analysis across seventeen HCs revealed two to three major components between 0-300 ms under both Face and Non-Face conditions. The subtraction analysis between these conditions demonstrated that while most components were mutually cancelled, the M120 component remained prominent, corresponding to cognitive differences between conditions. Similarly, CP showed three to five components under both conditions, with subtraction analysis highlighting the preserved M120 component. Detailed analysis of the waveforms, as depicted in Fig. 2 (left panels), revealed distinct patterns between HCs (upper panel) and CP (lower panel). The waveforms were color-coded according to channel locations indicated at the top left of each panel.

Analysis of frequency properties (theta: 4–7 Hz, alpha: 8–13 Hz, beta: 14–39 Hz, gamma: 40–98 Hz) revealed distinct group differences. As illustrated in Fig. 2 (right panels), HCs showed stronger suppression during Face conditions than Non-Face conditions across all frequency bands (latencies; theta: 450-500 ms, alpha: 200-500 ms, beta: 300-400 ms, gamma: 200-300 ms), while CP exhibited almost comparable responses without notable differences between conditions (green: Non-Face, red: Face, thin lines: ± SEM).

### Time–Frequency Representations (TFR): fig. 3

**Fig. 3:**
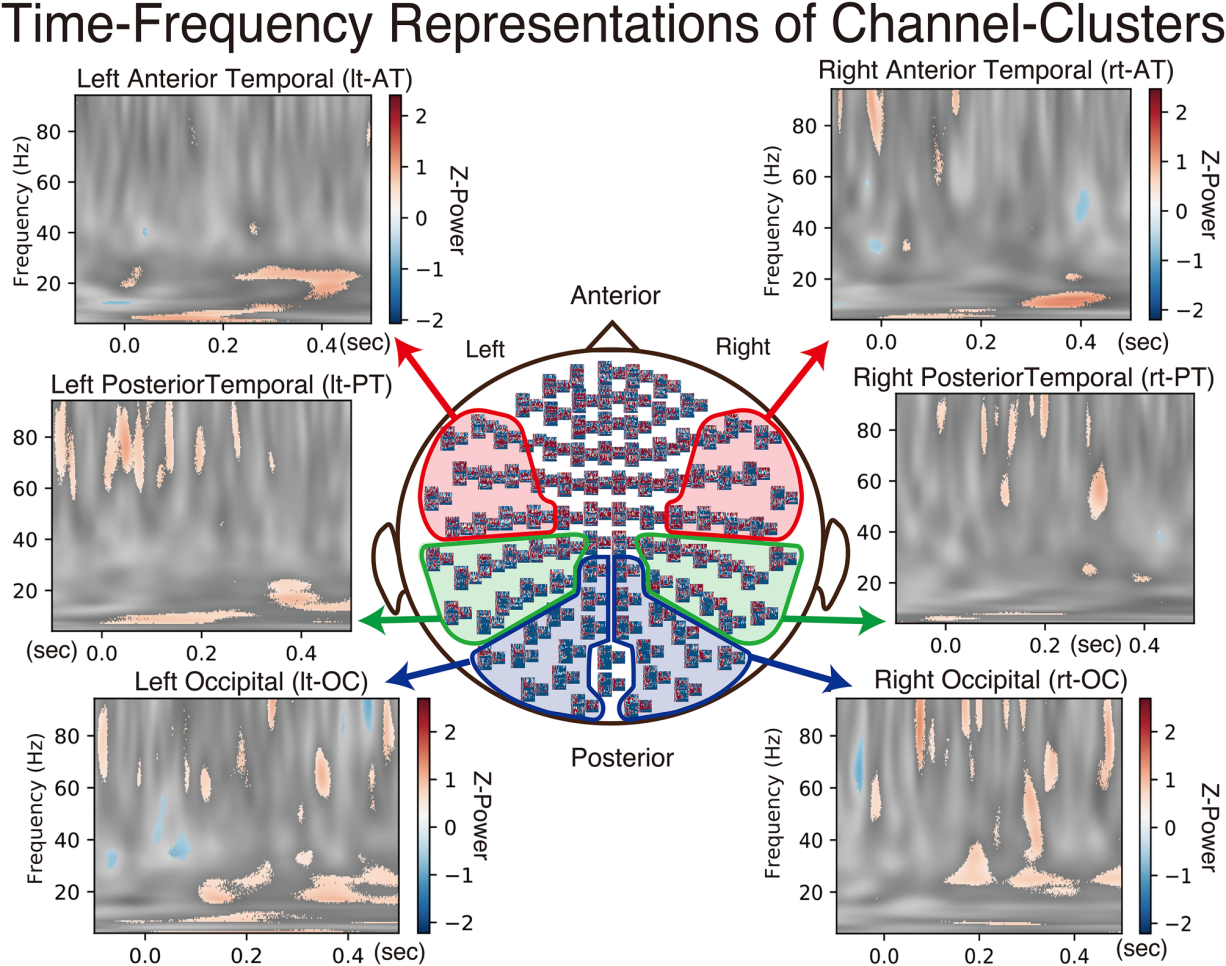
Time–frequency representations (TFR) comparing Face minus Non-Face responses between CP and HCs. TFRs were computed by first subtracting Non-Face from Face responses within each group, then comparing these difference scores between CP and HCs. The x-axis represents latency and the y-axis represents frequency, with the color bar indicating Z-power differences where red denotes stronger CP/Face responses and blue indicates stronger HC/Non-Face responses. Six channel-clusters were independently analyzed using 11 sensor locations each. Statistical significance was determined using cluster-based permutation testing (1000 permutations, p < 0.05), with only significant time–frequency clusters displayed in color and non-significant regions masked in gray. The primary significant finding was enhanced alpha-band activity (8–13 Hz) in the right anterior temporal channels (rt-AT) extending from approximately 300–450 ms, representing the sole effect that survived multiple comparison correction.

To clarify frequency-specific differences between groups and conditions, Face-minus-Non-Face difference maps for each participant were evaluated with a cluster-based permutation testing (1000 permutations, two-tailed, cluster threshold P < 0.05) using eleven-channel-clusters from bilateral occipital (OC), posterior temporal (PT), and anterior temporal (AT) channels. The differences were described as time–frequency representations (TFR) computed by first subtracting Non-Face from Face responses within each group, then comparing these differences between CP and HCs. Analysis was conducted over the full 4–90 Hz range, with the significance mask displayed in Fig. 3 to highlight the face-processing relevant frequency bands.

After applying appropriate statistical correction for multiple comparisons, only limited findings achieved statistical significance. In the alpha band (8–13 Hz), significant CP > HCs activity was observed in a right-lateralized AT cluster extending from approximately 300–450 ms (cluster p < 0.05, permutation corrected). This effect reflects weaker alpha suppression in CP relative to HCs and represents the primary finding that survived rigorous multiple comparison correction.

Other channel locations showed descriptive patterns but did not achieve statistical significance. The bilateral PT channels showed some enhancement around 120–240 ms, while OC channels displayed scattered clusters in the beta band (14–22 Hz) from approximately 200–400 ms. However, these patterns did not survive cluster-based permutation correction for multiple comparisons. In the theta (4–7 Hz) and gamma (> 30 Hz) bands, only scattered, spatially inconsistent clusters reached the uncorrected significance threshold with none forming coherent patterns, and no systematic CP–HCs differences were inferred after appropriate statistical correction.

### Band-specific time-course analysis (alpha-range for fig. 4)

**Fig. 4:**
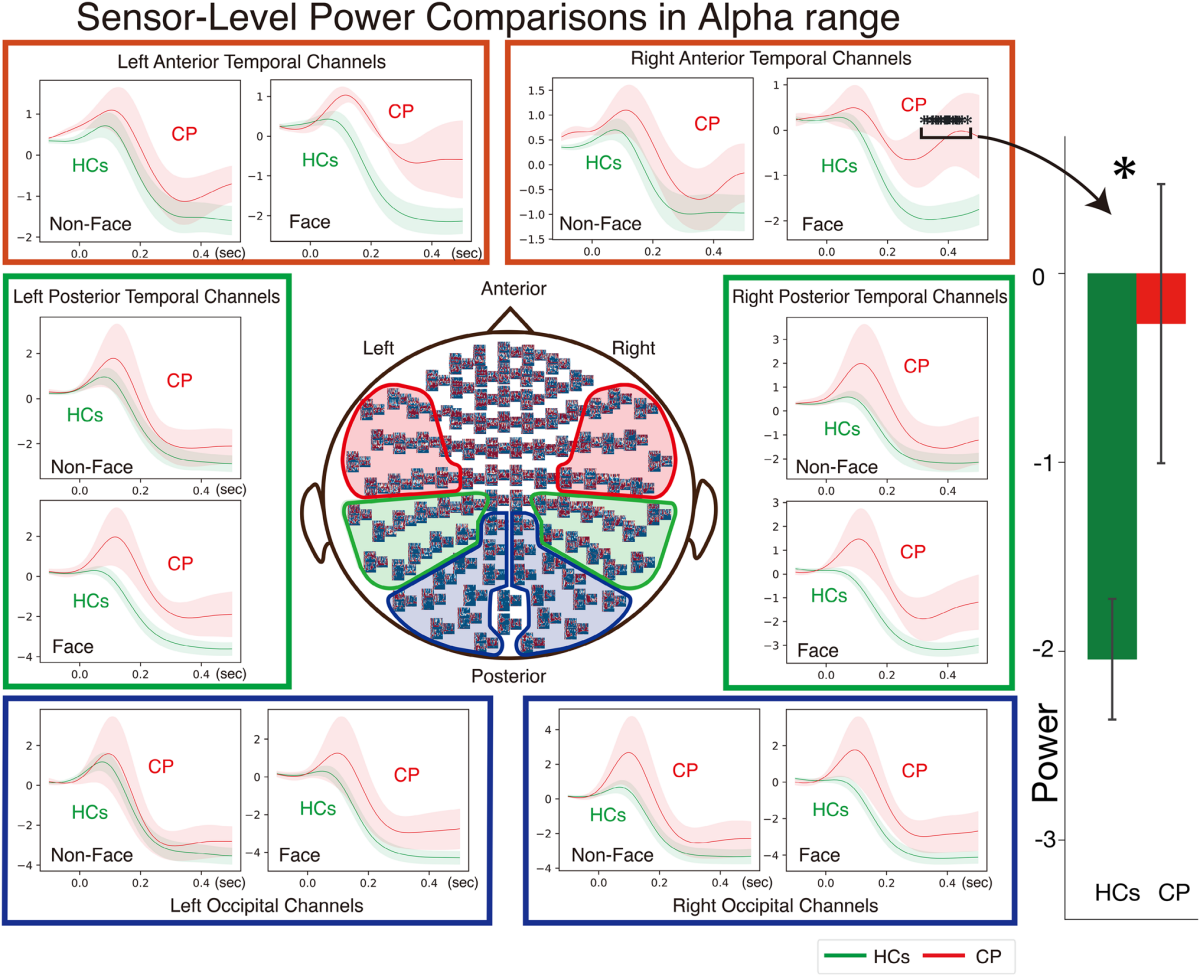
Sensor-level power comparisons between CP and HCs in Alpha range. The sensor-level response power of alpha frequency range was calculated from eleven locations of anterior temporal (AT), posterior temporal (PT), and occipital (OC) channels in each condition. The closed panels indicate Face or Non-Face conditions by comparing between CP and HCs in the same cortical channels. The green line denotes HCs while red line depicted CP with the thin line demonstrating ± 1 standard errors (SEM). Response differences between CP and HCs appeared only in Face conditions at the anterior channels in CP, which maximized at the right AT channels in the 300–450 ms time window. Asterisks indicate single latency windows of interest (≈ 300–450 ms) where permutation tests revealed statistically significant differences.

For each canonical band (theta 4–7 Hz, alpha 8–13 Hz, beta 14–38 Hz, gamma 40–98 Hz), power was averaged across predefined channel clusters and submitted to point-wise permutation testing (1000 label shuffles, two-tailed, P < 0.05) at every 1-ms sample between 0 and 500 ms.

After applying rigorous statistical correction across all frequency bands and channel locations, only the alpha band (8–13 Hz) yielded statistically significant between-group differences. Specifically, a single significant cluster (approximately 300–450 ms) was identified in the right AT channels. To quantify this effect, alpha power was averaged across the 300–450 ms interval and compared between groups using permutation testing, which confirmed a significant CP > HCs difference (P = 0.046).

All other frequency bands failed to achieve statistical significance after multiple comparison correction: Beta band (14–38 Hz) showed numerically weaker suppression over OC sensors but no time bins survived permutation testing (all p > 0.05, FDR-corrected); theta (4–7 Hz) and gamma (40–98 Hz) bands showed largely overlapping time-courses between groups with no significant differences at any channel cluster.

Given that only the alpha band survived statistical correction, Fig. 4 displays exclusively the alpha-band time-courses to highlight the sole validated finding. This represents the only statistically reliable CP-versus-HCs difference across all tested frequency bands, channel locations, and time points—specifically a late (300–450 ms) reduction of alpha suppression in right AT channels.

## Discussion

The present study investigated the neural mechanisms underlying face processing deficits in congenital prosopagnosia (CP) using magnetoencephalography (MEG) during a seeing-as-face (SAF) task. By comparing three individuals with CP with seventeen healthy controls (HCs), we identified neural signatures of face processing deficits in CP through frequency-specific abnormalities during face perception. Key finding emerged as enhanced alpha-band activity (8–13 Hz) at the channel-cluster covering the right anterior temporal in the 300–450 time window exclusively during face processing. This finding can be interpreted within the established framework of “core” and “extended” face processing networks^31,41^. However, our findings must be interpreted with significant caution given fundamental methodological limitations, including (1) the age range discrepancy between CP (22–31 years) and control groups (19–48 years), (2) use of archival control data without systematic diagnostic testing, (3) no power analysis to determine appropriate sample sizes, and (4) minimal CP sample size that severely limits statistical power and generalizability.

The preservation of the M120 component aligns with previous findings showing intact “core” scalp locations in CP^42,43^. The M120, originating from right extrastriatal cortices^17,18^, reflects preserved face categorization processes. However, the subsequent frequency-specific abnormalities in sensor-level measurements suggest altered neural processing patterns that may reflect disrupted information integration, though source reconstruction would be required to determine the specific brain networks involved. The pronounced alpha-band (8–13 Hz) abnormalities in the right anterior temporal channels at in the 300–450 ms time window is particularly significant, as ventral anterior temporal lobe (vATL) serves as a critical node in face identification networks^27^, though source reconstruction would be required to determine whether our sensor-level findings reflect activity from this specific brain region. In typical face processing, alpha-band suppression reflects active cortical processing and information integration^44^. Therefore, the enhanced activity in CP suggests disrupted normal information processing in these scalp locations. While the temporal characteristics (between 300–450 ms) and anterior temporal location of these abnormalities have been associated with face identity processing in previous literature^27,45^, our task design does not allow us to definitively separate identity processing from structural encoding components.

These patterns support conceptualizing CP as a disconnection syndrome^46^. Diffusion tensor imaging has demonstrated reduced structural integrity in white matter tracts connecting ventral occipitotemporal cortices (VOTC) to anterior temporal regions in CP^32–34^. Our findings of frequency-specific abnormalities may reflect this compromised connectivity, manifesting as insufficient alpha suppression in the right anterior temporal sensor locations. While previous studies suggest this scalp region may reflect activity from ventral anterior temporal areas, source reconstruction would be necessary to confirm the specific brain structures involved.

Several limitations of the current study warrant considerations. First, our sample size of three individuals with CP, unlike recent studies of more than a dozen cases^32,34^, limits the generalizability given CP’s heterogeneous presentation and ambiguities in diagnostic criteria^35,47–49^. Most critically, after correcting for multiple comparisons, only a single finding (alpha-band activity in right anterior temporal channels) achieved statistical significance, highlighting the preliminary nature of our observation. This minimal sample size is particularly problematic given the poor statistical power and inability to account for individual variability, such as different types of M170 activations^38^. Additionally, we did not conduct formal power analyses to determine appropriate sample sizes for either CP participants or controls, which represents a fundamental methodological limitation that affects the interpretability of our statistical comparisons. Our Japanese participants were identified using culturally adapted diagnostic tasks rather than globally standardized CP assessments, such as Cambridge Face Memory Test or PI20^50–52^, which may limit cross-cultural comparability. Furthermore, age differences between CP (22–31 years) and control groups (19–48 years), plus the use of archival control data without face processing verification may confound between-group comparisons. Moreover, our single-task design fails to provide convergent evidence across multiple face processing paradigms. The combination of small sample size, culturally specific diagnostics, inadequate control group characterization, and limited task battery substantially undermines the reliability and interpretability of our findings. Second, while our use of schematic faces allowed effective control for low-level visual properties and examination of early face categorization processes^18^, the absence of real faces may limit generalizability of our findings to real-world face recognition deficits. The “seeing object as a face” manipulation may emphasize top-down cognitive processes more than real faces. We acknowledge that without source reconstruction, we cannot make definitive claims about underlying brain regions and our findings reflect sensor-level measurements only. Third, our cross-sectional design limits our ability to distinguish whether the observed frequency-specific alterations represent primary deficits or compensatory mechanisms. Since prosopagnosia is a congenital condition, individuals with CP likely develop compensatory strategies over time, potentially modifying their symptoms and neural responses. The frequently reported difficulty in focusing on facial representations might reflect compensatory processing strategies rather than primary deficits. Future studies should address these limitations through larger cohorts that account for CP’s heterogeneity, with improved experimental designs incorporating both schematic and real faces. Our MEG data could support source reconstruction analysis, which would significantly enhance interpretation of our findings. However, the absence of individual structural MRI data for all participants (particularly our archival control dataset) prevents reliable source localization. Future studies should incorporate simultaneous MEG-MRI acquisition to enable precise source reconstruction and improve spatial specificity of findings. Longitudinal investigations tracking neural alterations and compensatory mechanisms could help distinguish primary deficits from compensatory processes and clarify the relationship between local neural abnormalities and global face processing deficits. These approaches could advance our understanding of CP’s neural basis and potentially inform more effective interventions.

Despite limitations, our findings have several important implications for understanding CP and face processing more generally. The temporal dissociation we observed between preserved early detection (M120) and impaired later processing supports a complex model of face processing^13^. Our findings extend this model by highlighting the importance of frequency-specific neural dynamics in different processing stages. The right anterior temporal abnormalities in the 300–450 time window suggest that this location plays a crucial role in face identification that is distinct from the initial structural encoding in core face processing regions^27,45^. The identification of specific frequency-band abnormalities provides potential neural signatures for CP, particularly the enhanced alpha-band activity in anterior temporal channels. These markers could potentially aid in earlier diagnosis and more precise characterization of CP subtypes, though validation in larger cohorts is essential.

## Conclusions

The present MEG study revealed distinct neural signatures of face processing deficits in congenital prosopagnosia through the application of the seeing-as-face task. Our findings demonstrate a pattern of preserved and impaired processing stages, characterized by the key frequency-specific abnormality: Enhanced alpha-band activity (8–13 Hz) in the right anterior temporal channels in the 300–450 time window. This abnormality was specific to face processing conditions, with preserved early face detection mechanisms as evidenced by intact M120 responses.

The temporal and spatial specificity of these sensor-level abnormalities suggests altered neural oscillations in CP during face processing. The enhanced alpha-band activity in the right anterior temporal channels likely reflects impaired face identity processing. This is consistent with previous structural connectivity studies suggesting CP as a disconnection syndrome^32,46^, though our sensor-level findings cannot directly demonstrate connectivity alterations.

These findings advance our understanding of the neural mechanisms underlying CP and identify frequency-specific neural patterns that require validation in larger cohorts before any clinical applications can be considered. Future research with adequately powered samples (based on formal power analyses), standardized diagnostic criteria, and investigation of functional connectivity will be essential to validate these preliminary observations and determine their broader applicability to the CP population. Longitudinal studies tracking neural alterations and compensatory mechanisms could help distinguish primary deficits from adaptive processes. This work contributes to a more comprehensive understanding of face processing networks and their dysfunction in developmental disorders.

## Methods

### Subjects

Seventeen healthy controls (HCs) (eleven women; mean age: 28.95 years; range: 19–48 years) and three individuals with congenital prosopagnosia (CP) (one woman; mean age: 26.3 years; range: 22–31 years) participated in this study. All participants had normal or corrected visual acuity and were right-handed as assessed by the Edinburgh Handedness Inventory^53^. In the process of reporting these three individuals of CP, strict measures were taken to ensure the confidentiality and privacy. The study was approved by the ethics committee of the Gunma University Hospital and conducted according to the Declaration of Helsinki. All participants provided written informed consent.

#### HC Participants

HCs’ data were derived from raw MEG data used in our previously published study^18^. Informed consent was obtained at the time of original measurement, with explicit written consent for use of their data in subsequent face recognition studies. During the development of our face recognition task, we confirmed that these participants showed no evidence of face recognition difficulties in social contexts. However, we acknowledge that detailed neuropsychological testing using standardized diagnostic measures, such as Cambridge Face Memory Test (CFMT) or the 20-item prosopagnosia index (PI20)^50–52^, was not conducted for the control group, which represents a significant limitation in confirming normal face processing abilities according to current field standards.

#### CP Participants

Individuals with CP were recruited from outpatient’s clinic and diagnosed by two experienced senior psychiatrists following comprehensive neuropsychological assessments. We acknowledge that our diagnostic approach used culturally adapted tasks rather than internationally standardized CP assessments (CFMT, CFPT, PI20), which limits direct comparison with international CP literature^36,49^. Our Japanese participants were identified using culturally adapted diagnostic tasks. This approach creates uncertainty about diagnostic equivalence across populations and reduces confidence in CP diagnosis according to current field standards.

Individuals with CP showed no abnormalities on memory, spatial organization, frontal function, procedural memory, and intelligence including the Wechsler Adult Intelligence Scale-Revised (WAIS-R), the Drawing Picture Recognition task, or the Cup Picture Recognition task, which involve pictures from a single category. However, the face picture recognition task revealed their specific impairment in face identification. This face-specific deficit pattern is consistent with previous findings showing dissociations between face and object recognition in CP^54^. CP showed selective impairment in face recognition (65.7% accuracy; healthy controls: 92.0%) while maintaining normal performance in object recognition belonging to one category (cups: 82.3% vs 87.7%; drawings: 92.3% vs 94.7%). Detailed neuropsychological assessments are shown in Table 1. Autism spectrum disorders were excluded through autism quotient scores and clinical assessment.

**Table 1:** The results of neurological examination of three individuals with CP.

		Case 1	Case 2	Case 3
Age		26 y/o	31 y/o	22 y/o
Gender		male	female	male
WAIS-R	FIQ	120	110	115
	VIQ	116	107	120
	PIQ	119	112	105
WAIS-III	FIQ	*	113	*
	VIQ		113	
	PIQ		110	
Face picture recognition (%) (Average of healthy control = 92.0)		67	63	67
	Number of recognize	9/15	8/15	10/15
	False negative	6	7	10
	False positive	4	4	0
Cup picture recognition (%) (Average of healthy control = 87.7)		83	87	77
	Number of recognize	12/15	11/15	12/15
	False negative	3	4	7
	False positive	2	2	0
Drawing picture recognition (%) (Average of healthy control = 94.7)		100	90	87
	Number of recognize	15/15	15/15	13/15
	False negative	0	0	4
	False positive	0	0	0

*, data missing; WAIS-R, Wechsler adult intelligence scale-revised; WAIS-III, Wechsler adult intelligence scale-third edition.

Table 1: The detailed results of neurological examinations adopted to three individuals with congenital prosopagnosia. All individuals exhibited moderate-to-superior intellectual skills with adequate attention, memory, and executive functions except for human face recognition. Although the neurological results on picture recognition of those belonging one category (e.g. cups) were normal (82.3% comparing with healthy control of 87.7%), the rate on face picture recognition was declining (65.7% comparing with healthy control of 92.0%).

#### Critical limitation

We acknowledge fundamental methodological limitations:

1. the age range discrepancy between CP (22–31 years) and control groups (19–48 years),
2. use of archival control data without systematic diagnostic testing, (3) no power analysis to determine appropriate sample sizes, and (4) minimal CP sample size that severely limits statistical power and generalizability.

### Stimuli

#### Stimuli and task

We employed the"seeing-as-face task"(SAF)^18^ consisting of two experimental blocks that presented identical sets of monochrome images containing four rectangles and four ovals (Fig. 1). In the first block (Non-Face condition), participants focused on rectangles, judging whether their sizes and locations matched the previous image. After confirming participants perceived these as abstract patterns, the second block (Face condition) instructed them to focus on the ovals arranged as facial features (two eyes, nose, and mouth) and judge whether successive face patterns were identical.

#### Task performance monitoring

Participants responded to each stimulus by pressing a button to indicate whether the current image matched the previous one. This design ensured sustained attention to the target features and provided behavioral confirmation of task engagement. After completing the Non-Face condition, both HC and CP participants described rectangle arrangements as"bones,""wall stains,“or”cow patterns."Subsequently, both groups were instructed that focusing on ovals would reveal face-like appearances, and several example images were presented before transitioning to the Face condition. All HC and CP successfully identified the face-like configurations in the Face condition. Notably, CP participants demonstrated preserved ability to detect schematic faces and perform the 1-back task, consistent with intact basic face detection processes in CP. No perceptual switching between“face”and"bone-like"interpretations was observed within the 500 ms presentation duration.

#### Stimulus timing and control

Each stimulus was presented for 500 ms with a 2500 ms inter-stimulus interval. Blocks used identical visual stimuli presented in the same sequence, differing only in participants’ perceptual interpretation. This design allowed extraction of face-specific cognitive processes by subtracting Non-Face from Face condition responses while controlling for low-level visual properties. Each condition comprised 200 trials, with total administration time of 20 min. The task order was fixed across all participants (Non-Face block followed by Face block) to ensure consistent perceptual priming effects.

### MEG recordings

MEG measurements were performed in a magnetically shielded room (JFE Mechanical Co., Tokyo, Japan) using a 306-channel whole-scalp neuromagnetometer (Vectorview, Elekta Neuromag, Helsinki, Finland). The system contained 102 measurement locations, each equipped with two orthogonal planar gradiometers and one magnetometer arranged in a helmet-shaped array. Participants maintained fixation on a central point with eyes open during stimulus presentation.

Head position was monitored using three fiduciary points (nasion and bilateral auricular points) and four head position indicator coils, digitized using an Isotrak three-dimensional digitizer (Polhemus™, Colchester, VT, USA). MEG responses were recorded with a 0.1–200 Hz bandpass filter at a 997 Hz sampling rate. Analysis periods included 400 ms with a 100 ms pre-stimulus baseline. Epochs exceeding 1500 fT/cm were excluded and replaced with additional recordings.

### Preprocessing and data analysis

Initially, all off-line analyses were based on the saved continuous raw data. For noise suppression and motion correction, the data were spatially filtered using the signal space separation method^55,56^ with Elekta Neuromag Maxfilter software, which suppresses noise generated by sources outside the brain. A notch filter was applied to eliminate noise from the power line (50 Hz) and its harmonics (100 Hz and 150 Hz). We eliminated eye movement- and body movements- related artifacts from the raw data using independent component analysis (ICA). The ICA was applied to the MEG sensor signals, and the eye movement and body movement signals were isolated based on visual inspection by two expert researchers. MEG data were then averaged separately according to their conditions (Non-Face and Face Conditions), obtaining two different epoched data sets. The differences between Non-Face and Face conditions were calculated by subtracting Non-Face from Face conditions formed as Subtraction at each channel of each digitized point. The mean amplitude from −100 to 0 ms before the onset of the stimulus was set as the baseline for each channel.

### Sensor-level analysis

MEG signals were separately averaged by time-locked triggers supplied at the initiation point of the visual presentations, which formed individual averaged data sets according to the conditions (Non-Face and Face conditions). Subtracted waveforms derived from the subtraction of the Non-Face condition from the Face condition were calculated for every subject. Epochs were rejected if the peak-to-peak amplitude during the target epoching period exceeded 400 fT and 4,000 fT/cm in any of the magnetometer and gradiometer channels, respectively. We calculated the differences at each digitized point to determine the difference between the two conditions (Face minus Non-Face) and the cognitive processes they tested. In estimating the subtraction waveforms, the mean amplitude from −100 ms to 0 ms was used as the baseline.

To analyze the magnetic signals, the amplitude of sensor-level field power was calculated from bilateral anterior temporal (AT), posterior temporal (PT) and occipital (OC) channel-clusters, which contains eleven channels. AT channels covered the local cerebral activities of anterior temporal activities, PT channels covered occipital to posterior temporal scalp locations, and OC channels covered posterior brain scalp locations. Importantly, we acknowledge that without source reconstruction, we cannot make definitive claims about underlying brain regions and our findings reflect sensor-level measurements only. Those sensor-level activity patterns were compared between two conditions and groups. Corresponding to the results of waveform analysis, total and channel powers of the waveform were calculated by the square root of the sum of squared fT/cm values corresponding to the frequency of theta (4-7 Hz), alpha (8-13 Hz), beta (14-39 Hz), and gamma (40-98 Hz) in each condition. All calculations were adopted using the minimum norm estimates (MNE) suite and MNE python, which are widely used distributed source models for MEG analysis (http://www.martinos.org/mne/)^57^. Time–frequency representations (TFR) were calculated by subtracting Non-Face from Face conditions waveform power according to frequency (8-25 Hz) and latency (−100 to 500 ms) using Morlet wavelets.

### Statistical analysis

All inferential tests were fully non-parametric and implemented in Python (NumPy 1.26, SciPy 1.12, statsmodels 0.14).

#### Time-frequency map

For every region-of-interest (ROI) we averaged power across the constituent channels and compared CP with HCs at each time-sample (0—500 ms) and frequency bin (4—98 Hz). Statistic. Difference of group means.

Permutation test. Group labels were randomly reassigned 1000 times; the two-tailed P-value equals the proportion of permuted statistics that exceeded the observed value.

Display. Bins with P < 0.05 are rendered as colored overlays on the grey-scale TFR (Fig. 3).

#### Band-averaged time courses

Canonical bands were defined as theta 4–7 Hz, alpha 8–13 Hz, beta 14–38 Hz, and gamma 40–98 Hz. Power was first averaged across the frequencies in each band and then submitted to the same point-wise permutation test (1000 shuffles, two-tailed) at every 1-ms sample. Significant samples (P < 0.05) are marked by asterisks in Fig. 4.

#### Interval-mean comparison (alpha 300–450 ms)

To quantify the late alpha effect over the right anterior temporal channels, power within 300–450 ms was averaged for each participant and re-tested with the permutation procedure.

The permutation test is non-parametric (no assumptions about normality or equal variance). Because statistical inference was restricted to four predefined frequency bands and a single latency window of interest (300–450 ms), no further multiple-comparison correction was applied; the per-window permutation test therefore controls the family-wise error within each tested window.

## Data Availability

Analysis codes are made publicly available through GitHub ([https://github.com/Magnetoencephalography/C/_P] (https://github.com/Magnetoencephalography/C_P)) and permanently archived with DOI via Zenodo (10.5281/zenodo.16749552). Raw individual MEG data and associated processing files are not publicly accessible because our original institutional review board (IRB) approval and informed consent procedures did not include provisions for open public data sharing when this study was conducted. However, these data may be shared with qualified researchers upon reasonable request to the corresponding author (Dr. Yutaka Kato, yutaka.kt@gunma-u.ac.jp), subject to appropriate data sharing agreements, anonymization procedures, and institutional approval. All processed datasets and statistical analyses presented in this study are included within the manuscript. MEG data preprocessing and analysis were conducted using the open-source MNE-Python software suite, available at [https://mne.tools/stable/index.html] (https:/www.martinos.org/mne). Future studies will incorporate comprehensive data sharing provisions in ethical approvals to support evolving standards of open science practices.
